# Utility of gene methylation analysis, cytological examination, and HPV-16/18 genotyping in triage of high-risk human papilloma virus-positive women

**DOI:** 10.18632/oncotarget.19459

**Published:** 2017-07-22

**Authors:** Yan Tian, Na-Yi Yuan Wu, Yu-Ligh Liou, Ching-Tung Yeh, Lanqin Cao, Ya-Nan Kang, Huei-Jen Wang, Yichen Li, Tang-Yuan Chu, Wei Li, Xiang Liu, Yi Zhang, Honghao Zhou, Yu Zhang

**Affiliations:** ^1^ Department of Obstetrics & Gynecology, Xiangya Hospital, Hunan, P. R. China; ^2^ Department of Clinical Pharmacology, Xiangya Hospital, Central South University, Changsha, P. R. China; ^3^ Institute of Clinical Pharmacology, Central South University, Hunan Key Laboratory of Pharmacogenetics, Changsha, P. R. China; ^4^ iStat Biomedical Co. Ltd., Taipei, Taiwan; ^5^ Department of Obstetrics & Gynecology, Buddhist Tzu Chi General Hospital, Hualien, Taiwan; ^6^ Department of Obstetrics and Gynecology, ShengJing Hospital of China Medical University, Liaoning, P. R. China; ^7^ Department of Pharmacy, Xiangtan Central Hospital, Xiangtan, P. R. China

**Keywords:** HPV triage, PAX1, ZNF582, HPV16/18 genotyping, cervical cancer

## Abstract

In 2015, the American Society for Colposcopy and Cervical Pathology and the Society of Gynecologic Oncology issued interim guidance for the use of a human papillomavirus (HPV) test for primary screening, suggesting triage of women positive for high-risk human papillomavirus (hrHPV) by HPV-16/18 genotyping and cytology for women positive for non-16/18 hrHPV. The design of the present study was based on this interim guidance and analysis of the methylation status of specific candidate genes, which has been proposed as a tool to reduce unnecessary referral following primary HPV screening for cervical cancer. We performed a hospital-based case-control study including 312 hrHPV-positive women. hrHPV genotyping was performed by nested multiplex PCR assay with type-specific primers.Residual cervical cells from liquid-based cytology were used for extraction of genomic DNA for assessment of the methylation status of *PAX1*, *ZNF582*, *SOX1*, and *NKX6-1* and HPV genotyping. Combined with HPV-16/18 genotyping, both a dual methylation test for *PAX1*/*ZNF582* and testing for *ZNF582* methylation demonstrated 100% association of methylation with pathology results, indicating carcinoma *in situ* or squamous cell carcinoma. The sensitivity and specificity of the dual methylation test for *PAX1*/*ZNF582* as a reflex test for identification of CIN3+ lesions were 78.85% and 73.55% (odds ratio = 10.37, 95% confidence interval = 4.76–22.58), respectively. This strategy could reduce the number of patients referred for colposcopic examination by 31.3% compared with cytology, and thus provide a feasible follow-up solution in regions where colposcopy is not readily available. This strategy could also prevent unnecessary anxiety in women with hrHPV infection.

## INTRODUCTION

Infection with a human papillomavirus with a high-risk genotype (hrHPV) is the only known etiologic risk factor for the development of cervical cancer and a necessary step in carcinogenesis [1–4]. The development of cervical cancer is strongly associated with previous infection with hrHPV, and cervical cancer represents 53.4% of all hrHPV-associated cancers in women [5]. Direct referral of HPV16/18-positive women for colposcopy by primary hrHPV screening is a clinical practice in the USA and will be standard clinical practice in both Australia and New Zealand soon; other countries adopting primary HPV screening in the future will likely also adopt this partial genotyping protocol [6–9].

The American Cancer Society (ACS) and the American Society for Colposcopy and Cervical Pathology (ASCCP) recommend that either co-testing every 5 years or cytology-only screening every 3 years should be implemented for women aged 30–65 years, whereas cytology alone should be performed for women between 21 and 30 years of age [9]. Several studies have shown that primary screening for hrHPV is more sensitive than cytology; however, it is less specific, and the resulting decreased positive predictive value (PPV) for CIN3 may lead to over-referral and overtreatment of patients [10–12]. Therefore, women with specimens testing positive for hrHPV require additional triage. An approach that has gradually gained favor for women aged over 25 or 30 years is the use of high-sensitivity hrHPV testing as the primary screening method, followed by triage of hrHPV-positive women by high-specificity Pap smear. In early 2015, the Society of Gynecologic Oncology and the ASCCP jointly issued interim clinical guidance regarding the use of primary testing for high-risk human papillomavirus for cervical cancer screening. The interim clinical guidance provides for the use of primary screening for hrHPV as a possible method for early cervical pre-cancer and cancer detection in the United States, based on academic guidance. The interim clinical guidance increases the number of cervical cancer programs in the United States from two to three [13,14].

In China, cervical cancer remains the seventh-leading cause of death from cancer among females, and there were almost 62,000 new cases and 30,000 deaths due to cervical cancer in 2012 [15]. In urban areas of China, cervical cancer diagnosis procedures are well established; however, awareness of cervical cancer screening is still deficient. In certain developed cities, such as Beijing and Shanghai, the incidence of cervical cancer has decreased significantly owing to the wide promotion of cervical cancer prevention information and opportunistic screening in hospitals. In a study of 37 cities in China, the total positive rate for hrHPV was 21.07%, ranging from 18.42% to 31.94% and varying by region [16]. From HPV genotyping studies, the most common HPV genotypes in Chinese women with cervical cancer are HPV-16, -18, -58, -33, and -52 [2]. It is difficult to achieve co-testing (i.e., HPV testing combined with cytology screening) or cytology testing as the primary screening method in China in the short term because of the lack of infrastructure to support such a screening program across the huge Chinese population.

The use of HPV testing has increased dramatically in China in recent years because of the achievement of consistent and repeatable results. The lack of clinical validation of the majority of HPV tests used in hospitals has led to confusion and huge numbers of women undergoing colposcopic examination. The aims of cervical cancer screening are to discover high-grade lesions or cancer, rather than to detect the virus, whose presence does not necessarily indicate malignant disease and can thus lead to unnecessary anxiety for the women tested. Therefore, there is an urgent need for an objective, reproducible, and accurate method of screening for cervical cancer in China.

One such screening possibility arises from the field of epigenetics [17]. Numerous investigations have reported that gene-specific hypermethylation, which occurs in the pre-invasive and invasive phases of cervical cancer, is a promising biomarker for early diagnosis [18,19]. Several studies examining the genes paired box 1 (*PAX1*), sex determining region Y-box 1 (*SOX1*), zinc finger protein 582 (*ZNF582*), and NK6 transcription factor-related locus1 (*NKX6-1*) have reported their potential as biomarkers for cervical cancer screening and for triage of cytological diagnoses and high-risk HPV infection groups [20–25]. In fact, methylated *PAX1* (*PAX1*^*m*^), *SOX1* (*SOX1*^*m*^), *ZNF582* (*ZNF582*^*m*^), and *NKX6-1* (*NKX6-1*^*m*^) have been extensively documented as highly sensitive biomarkers for detection of cervical intraepithelial neoplasia at grade 3 or higher (CIN3+) [26]. In particular, *ZNF582*^*m*^ has been found to have clinical potential for cancer detection. Clinically, *ZNF582*^*m*^ demonstrated 70% sensitivity and 82% specificity for CIN3+ lesions in a Taiwanese case-control cohort [27]. *ZNF582*^*m*^ also has good sensitivity and specificity for triage of low-grade squamous intraepithelial lesion (LSIL), with values of 73% and 71%, respectively [22]. Moreover, *ZNF582* has been reported to exhibit a high methylation rate in cervical adenocarcinoma, which has been difficult to diagnose [28].

Here we report a case-control study of diagnosis of CIN3+ in hrHPV-positive cases negative for HPV 16/18 by detection of methylated DNA, HPV genotyping, liquid-based cytology, and combinations thereof. The results were analyzed following the recommended primary HPV screening protocol algorithm [13]. The analysis procedure was as follows: hrHPV-positive patients were triaged through genotyping for HPV16/18 and reflex tests for the 12 hrHPV genotypes (excluding HPV-16/18). The results of the reflex tests, including testing of the methylation status of *PAX1*, *ZNF582*, *SOX1*, and *NKX6-1* and liquid Pap smears, were compared and analyzed. We propose that this approach would be useful in outpatient departments of hospitals in highly populated countries, where screening is not readily accessible through community-based settings.

## RESULTS

### Characteristics of subjects and colposcopic biopsy results

**Figure 1:**
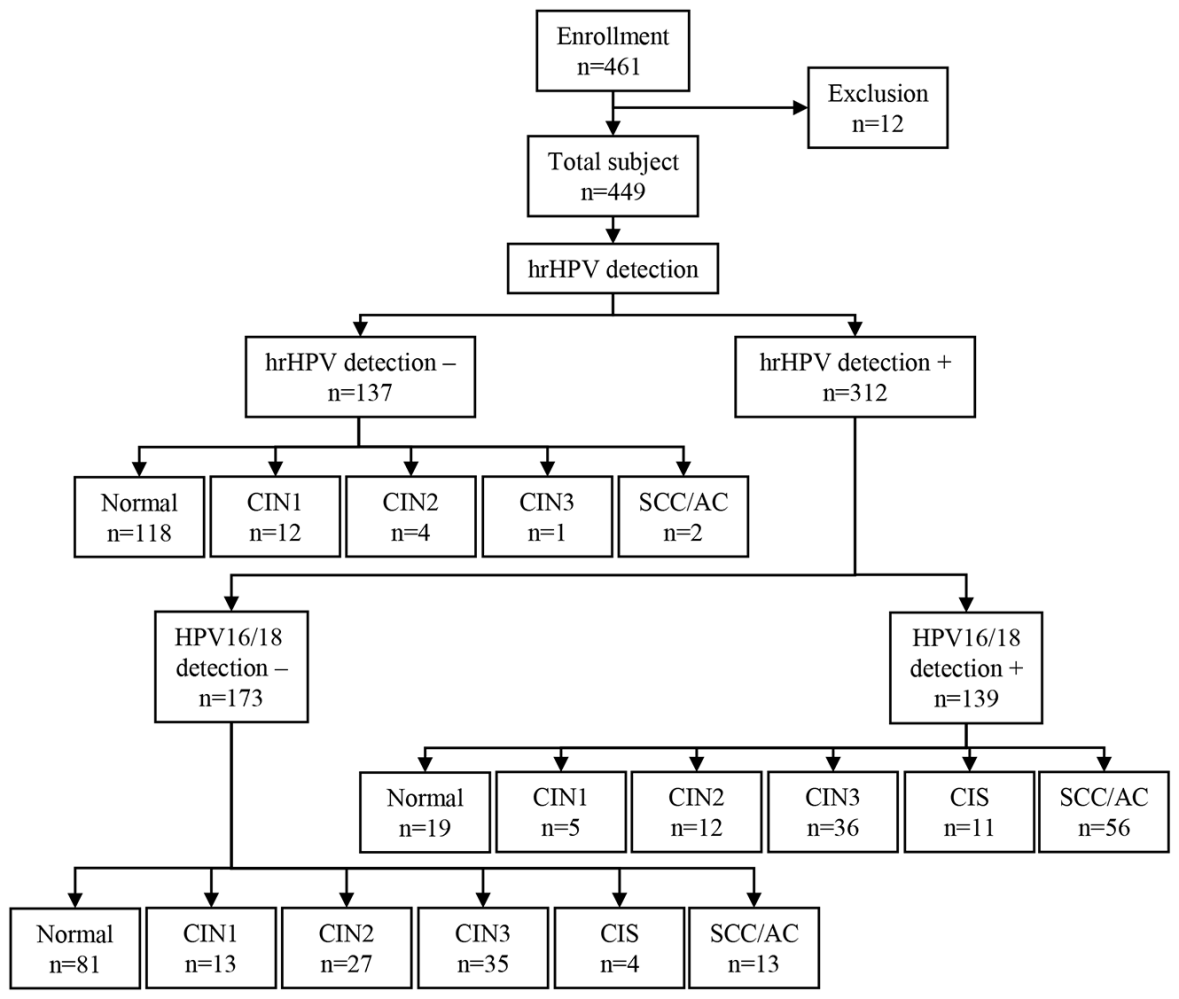
Study flow chart from enrollment to hrHPV outcome A total of 461 women participated in cervical cancer screening at the colposcopy examination room of the Department of Obstetrics & Gynecology, Xiangya Hospital. All women underwent colposcopy and biopsy. Histopathology diagnoses were used as endpoints for the analysis. Twelve women were excluded because they did not meet the inclusion criteria. CIN1, cervical intraepithelial neoplasia type 1; CIN2, cervical intraepithelial neoplasia type 2; CIN3, cervical intraepithelial neoplasia type 3; CIS, carcinoma in situ; SCC, squamous cell carcinoma; AC, adenocarcinoma.

In total, 461 patients were enrolled in this study, of which 12 were excluded, 137 were hrHPV-negative, and 312 were hrHPV positive (Figure 1). The overall sensitivity and specificity for identification of CIN3+ in hrHPV-positive patients were 98.10% and 46.05%, respectively. The percentage of HPV-16/18-positive hrHPV patients in each pathological category was as follows: normal (19.00%); CIN1 (27.77%); CIN2 (30.76%); CIN3 (50.70%); carcinoma in situ (CIS) (73.33%); and squamous cell carcinoma (SCC)/ adenocarcinoma (AC) (81.15%). The sensitivity of HPV16/18, *ZNF582*^*m*^, and *PAX1*^*m*^ */ZNF582*^*m*^ was 66.5%, 77.4% and 86.5% for CIN3+ lesions in the HPV positive patients (n=312), respectively. The specificity of HPV16/18, *ZNF582*^*m*^, and *PAX1*^*m*^ */ZNF582*^*m*^ was 77.1%, 77.7%, and 65.0%, respectively. The referral rate of HPV16/18, *ZNF582*^*m*^, and *PAX1*^*m*^ */ZNF582*^*m*^ was 44.6%, 50.0%, and 60.6%, respectively. There were 173 HPV-16/18-negative hrHPV patients, of whom 81 (46.82%) had normal pathologic results, 13 (7.51%) were CIN1, 27 (15.60%) CIN2, 35 (20.23%) CIN3, 4 (2.31%) CIS, and 13 (7.51%) SCC. The demographic characteristics, reflex testing results (including cytology), most common HPV genotypes in Chinese women, and methylation results for *PAX1*, *ZNF582*, *SOX1*, and *NKX6-1* are presented in Table 1 . In the cytology results for the hrHPV HPV 16/18-negative cohort, 149 (86.12%) subjects were classified at or above the atypical squamous cells of undetermined significance (ASC-US+) level, of which 93 were ASC-US, 6 LSIL, and 50 had higher grade lesions (ASC-H/AGC/HSIL+). The percentage of positive HPV genotypes determined by the reflex tests (alone or in combination with the non-16/18 types of hrHPV) was 32.37% for HPV-52, 26.59% for HPV-58, 10.40% for HPV-33, 8.09% for HPV-31, and 41.62% for HPV- 35/45/51/56/59/66/68. The positive rate for individual methylated genes in the CIN3+ group was 71.15%, 59.62%, 69.23%, and 59.62% for *PAX1*^*m*^, *ZNF582*^*m*^, *SOX1*^*m*^, and *NKX6-1*^*m*^, respectively.

**Table 1:** The distribution DNA methylated genes and HPV genotyping tests for a non-16/18 high risk type positive women

Cutoff	Histological results						Total	
	Normal	CIN1	CIN2	CIN3	CIS	SCC/AC		
**Number of subjects**								
N	81	13	27	35	4	13		173
%	46.82%	7.51%	15.61%	20.23%	2.31%	7.51%		100%
Age								
Mean ± SD	41.9 ± 10.0	43.0 ± 10.8	37.2 ± 11.2	42.1± 6.9	45.0 ± 7.5	52.2 ± 9.5		42.1 ± 10.1
(range)	(25.5 to 77.8)	(29.0 to 65.2)	(21.8 to 61.2)	(28.7 to 62.5)	(36.7 to 64.2)	(38.2 to 70.5)		(21.8 to 77.8)
**Cytology results**								
Normal	22	1	0	0	0	1		24
%	91.66%	4.17%	0.00%	0.00%	0.00%	4.17%		100%
ASC-US	50	8	22	12	0	1		93
%	53.76%	8.60%	23.66%	12.90%	0.00%	1.08%		100%
LSIL	1	2	0	3	0	0		6
%	16.67%	33.33%	0.00%	50.00%	0.00%	0.00%		100%
ASC-H/AGC/HSIL+	8	2	5	20	4	11		50
%	61.54%	15.38%	18.52%	57.14%	100.0%	84.62%		100%
**Detection modality or test used**								
HPV31	5	0	2	4	0	3		14
%	6.17%	0.00%	7.41%	11.43%	0.00%	23.08%		8.09%
HPV33	4	1	4	8	0	1		18
%	4.94%	7.69%	14.81%	22.86%	0.00%	7.69%		10.40%
HPV52	27	3	9	14	0	3		56
%	33.33%	23.08%	33.33%	40.00%	0.00%	23.08%		32.37%
HPV58	20	4	9	8	2	3		46
%	24.69%	30.77%	33.33%	22.86%	50.00%	23.08%		26.59%
HPV35/39/45/51/56/59/66/68	37	6	12	11	2	4		72
%	45.68%	46.15%	44.44%	31.43%	50.00%	30.77%		41.62%
*PAX1*^*m*^ ΔCp≦9.0	14	2	8	23	4	10		61
%	17.28%	15.38%	29.63%	65.71%	100.0%	76.92%		35.26%
*ZNF582*^*m*^ ΔCp≦11.0	10	3	3	14	4	13		47
%	12.35%	23.08%	11.11%	40.00%	100.0%	100.0%		27.17%
*SOX1*^*m*^ ΔCp≦8.0	12	5	4	21	4	11		57
%	14.81%	38.46%	14.81%	60.00%	100.0%	84.62%		32.95%
*NKX6.1*^*m*^ ΔCp≦11.0	27	7	7	21	3	7		72
%	33.33%	53.85%	25.93%	60.00%	75.00%	53.85%		41.62%

### Sensitivity and specificity of testing for CIN3+ by analysis of methylated genes, HPV genotyping, and cytology

The sensitivity, specificity, and AUC results for the different molecular reflex tests for detection of CIN3+ lesions, individually and in combination, are presented in Table 2 . Pap smears achieved 98.08% sensitivity and 19.01% specificity at the ASC-US cut-off value included in the interim clinical guidance. The AUC for HPV genotyping individually or combination was < 60% and the odds ratio (OR) was < 4. Three of the DNA methylation markers, i.e., *PAX1*^*m*^, *SOX1*^*m*^, and *ZNF582*^*m*^, used individually resulted in AUC > 75%; *ZNF582*^*m*^had the highest AUC at 79.0% (confidence interval (CI), 71.2%–86.7%). The sensitivity and specificity of *ZNF582*^***m***^ in the identification of CIN3+ lesions were 76.92% and 80.99%, respectively.

**Table 2:** The performance of PAX1^*m*^, ZNF582^*m*^, SOX1^*m*^, NKX6.1^*m*^ and HPV infection tests in detection of CIN3+ lesion

Target genes	Cutoff	Sensitivity (%) (95%CI)	Specificity (%) (95%CI)	PPV (%) (95%CI)	AUC (%) (95%CI)	Odds ratio (95%CI)	*P* value
Pap smear	≧ASCUS	98.08 (89.88-99.66)	19.01 (13.01-26.91)	34.23 (26.66-42.44)	58.5 (49.8-67.3)	11.97 (1.57-91.18)	0.002$
	≧ASC-H/AGC/HSIL+	67.31 (53.76-78.48)	87.60 (80.55-92.34)	70.00 (55.39-82.14)	77.5 (69.1-85.8)	14.55 (6.59-32.14)	<0.001
*PAX1*^*m*^	≦9.0	71.15 (57.73-81.67)	80.17 (72.18-86.29)	60.66 (49.31-72.93)	75.7 (67.4-83.9)	9.97 (4.72-21.06)	<0.001
*ZNF582*^*m*^	≦11.0	59.62 (46.07-71.84)	86.78 (79.60-91.69)	65.96 (50.69-79.14)	73.2 (64.3-82.0)	9.69 (4.51-20.80)	<0.001
*SOX1*^*m*^	≦8.0	69.23 (55.73-80.09)	82.64 (74.92-88.36)	63.16 (49.34-75.55)	75.9 (67.6-84.3)	10.71 (5.04-22.77)	<0.001
*NKX6.1*^*m*^	≦11.0	59.62 (46.07-71.84)	66.12 (57.30-73.94)	43.06 (31.43-55.27)	62.9 (53.7-72.0)	2.88 (1.47-5.63)	0.002
HPV31		13.46 (6.68-25.27)	94.21 (88.54-97.17)	50.00 (23.04-76.96)	53.8 (44.2-63.4)	2.53 (0.84-7.63)	0.090
HPV33		17.31 (9.38-29.73)	92.56 (86.47-96.04)	50.00 (26.02-73.98)	54.9 (45.3-64.6)	2.61 (0.97-7.00)	0.051
HPV52		32.69 (21.52-46.24)	67.77 (59.01-75.44)	30.36 (18.78-44.10)	50.2 (40.8-59.6)	1.02 (0.51-2.04)	0.953
HPV58		25.00 (15.23-38.21)	72.73 (64.18-79.87)	29.79 (17.34-44.89)	48.9 (39.5-58.2)	0.89 (0.42-1.87)	0.756
HPV31/33		30.77 (19.91-44.27)	88.43 (81.51-92.98)	53.33 (34.33-71.66)	59.6 (50.0-69.2)	3.40 (1.51-7.64)	0.002
HPV52/58		57.69 (44.19-70.13)	40.50 (32.17-49.40)	29.41 (20.80-39.25)	49.1 (39.7-58.5)	0.93 (0.48-1.79)	0.824
HPV31/33/52/58		84.62 (72.48-91.99)	29.75 (22.33-38.42)	34.11 (25.99-42.97)	57.2 (48.2-66.2)	2.33 (1.00-5.44)	0.047
HPV35/39/45/51/56/59/66/68		32.69 (21.52-46.24)	54.55 (45.67-63.14)	23.61 (14.40-36.09)	43.6 (34.4-52.8)	0.58 (0.30-1.15)	0.118
*PAX1*^*m*^ or *ZNF582*^*m*^		78.85 (65.97-87.76)	73.55 (65.06-80.60)	56.16 (44.05-67.76)	76.2 (68.3-84.1)	10.37 (4.76-22.58)	<0.001
*PAX1*^*m*^ or *SOX1*^*m*^		80.77 (68.10-89.20)	74.38 (65.94-81.32)	57.53 (45.41-69.03)	77.6 (69.9-85.3)	12.19 (5.47-27.18)	<0.001
*PAX1*^*m*^ or *NKX6.1*^*m*^		80.77 (68.10-89.20)	57.85 (48.94-66.28)	45.16 (34.81-55.83)	69.3 (61.0-77.6)	5.77 (2.65-12.56)	<0.001
*ZNF582*^*m*^ or *SOX1*^*m*^		75.00 (61.79-84.77)	76.86 (68.59-83.48)	58.21 (45.52-70.15)	75.9 (67.8-84.0)	9.96 (4.68-21.24)	<0.001
*ZNF582*^m^ or *NKX6.1*^m^		78.85 (65.97-87.76)	57.85 (48.94-66.28)	45.45 (35.41-55.77)	68.3 (59.9-76.8)	5.12 (2.4-10.91)	<0.001
*SOX1*^*m*^ or *NKX6.1*^*m*^		82.69 (70.27-90.62)	60.33 (51.43-68.60)	47.25 (36.69-58.00)	71.5 (63.4-79.6)	7.27 (3.25-16.26)	<0.001

*P* value determined by chi-square test or by Fisher’s exact test; CI, confidence interval; HPV, human papillomavirus; hrHPV, high-risk human papillomavirus; odds ratio for CIN3+; *Gene*^*m*^, methylated *Gene*.

Dual DNA methylation testing of positivity for either gene in pairs of *PAX1*^*m*^/*ZNF582*^*m*^, *PAX1*^*m*^/*SOX1*^*m*^, and *ZNF582*^*m*^/*SOX1*^*m*^ resulted in AUC of 76.2%, 77.6%, and 75.9%. The highest odds ratios for combinations of two methylated genes were those for *PAX1*^*m*^/*ZNF582*^*m*^ and *PAX*1^m^/*SOX1*^*m*^, at 10.37 and 12.19, respectively.

### Colposcopy referral rates: the rates of positivity for each test in the different histologic categories

**Figure 2:**
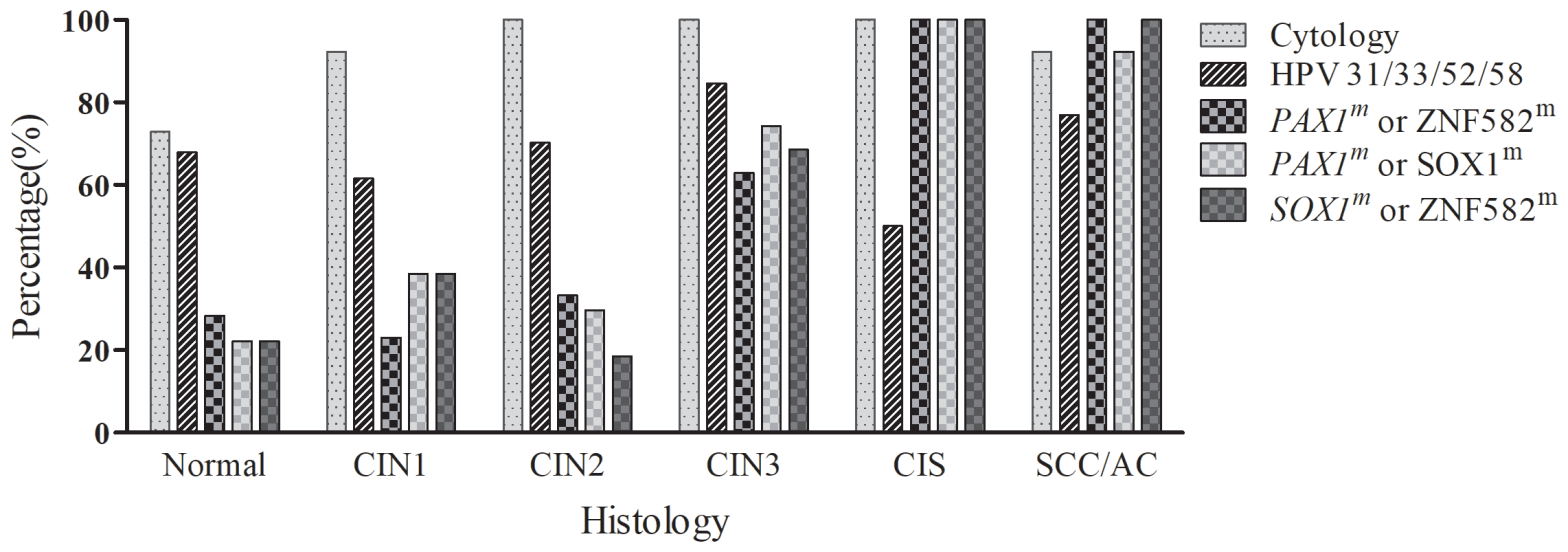
The percentage of positive findings in the pathologic categories using different individual or combined tests The bar chart shows the positive rate for each test in each histologic category. CIN1, cervical intraepithelial neoplasia type 1; CIN2, cervical intraepithelial neoplasia type 2; CIN3, cervical intraepithelial neoplasia type 3; CIS, carcinoma in situ; SCC, squamous cell carcinoma; AC, adenocarcinoma.

A bar chart showing the percentage of women with cervical changes in different histologic categories who tested positive by Pap smear or combinations of HPV genotyping and gene methylation tests is presented in Figure 2 (only the more effective genotype/methylation tests are presented). Analysis of the distribution of positive results for each histologic category demonstrated that Pap smears had very high positive rates in each category, HPV-31/33/52/58 genotype was associated with an 84.6% positive rate for CIN3+, in addition, with 70.2% positivity for CIN2-. The dual methylation markers *PAX1*^*m*^/*ZNF582*^*m*^, *PAX1*^*m*^/*SOX1*^*m*^, and *SOX1*^*m*^/*ZNF582*^*m*^ were the most discriminatory. *ZNF582*^*m*^ combined with either *PAX1*^*m*^ or *SOX1*^*m*^ achieved 100% positivity for histology results indicating cancer; however, the positive rate decreased to between 62.86% and 74.29% for the CIN3 category (Figure 2).

Colposcopy is a diagnostic procedure performed by physicians to examine the cervix, vagina, and vulva closely for signs of disease. Due to the high cost and insufficient availability of colposcopy, it is desirable to reduce the number of colposcopy referrals, while not missing women with severe dysplasia or carcinoma (CIN3, CIS, or SCC). Our results indicated that the referral rates for colposcopy after reflex tests using the individual methylated genes *PAX1*^*m*^, *ZNF582*^*m*^ and *SOX1*^*m*^ were 35.26%, 27.17%, and 32.95%, respectively, which was lower than the rate with cytology (86.1%; cut off at ASC-US) (Table 1). Using dual tests for methylated genes (*PAX1*^*m*^/*SOX1*^*m*^ or *PAX1*^*m*^/*ZNF582*^*m*^) as reflex tests, the positive rates for CIN3+ histology were 6.94% higher than for *PAX1*^*m*^ alone, and use of the *SOX1*^*m*^/*ZNF582*^*m*^ dual test increased positive rates for CIN3+ by 11.56% compared with *ZNF582*^*m*^ alone, as shown in Figure 2.

## DISCUSSION

HPV infection is a major cause of cervical cancer. HPV genotype testing, based on the identification of HPV DNA in the cervix, has the potential to improve HPV testing and demonstrates higher sensitivity for high-grade CIN than cytology testing, based on the identification of changes in cellular morphology. DNA testing for high-risk HPV types is frequently performed in parallel with cytology to detect high-grade dysplasia and cervical cancer, particularly in women over 30 years of age. HPV DNA testing is a relatively simple process and therefore advantageous for large-scale screening in China or developing countries, which are lacking cytology infrastructure and expert cytologists. In addition, HPV DNA testing of self-collected vaginal samples can be less uncomfortable for conservative women, and can increase participation rates in primary or routine screening for cervical cancer [29–32].

It is believed that only persistent HPV infections are associated with pre-cancerous lesions. Despite its high sensitivity, hrHPV testing cannot distinguish whether or not an HPV-positive result is associated with a clinically relevant lesion, and a positive hrHPV result may lead to over-interpretation of minor cellular abnormalities, thereby reducing the specificity of this technique [33]. Therefore, combination tests for methylated *PAX1*, *ZNF582*, and HPV16/18 that can distinguish between the CIN3+ and CIN2- groups are urgently required. Cytology is considered appropriate for application as a reflex test for hrHPV-positive women [34]. For detection of CIN3+, the sensitivity of triage testing by cytology alone was 65.8% (95% CI, 50.7–80.9) and the specificity was 78.6% (95% CI, 72.8–84.3) [35]. When using other technology for hrHPV triage, the sensitivity of p16/Ki-67 dual staining for ≥CIN3 was 93.8%, which did not differ significantly from the results obtained from Pap cytology (87.7%). The specificity of p16/Ki-67 dual staining for ≥CIN3 was 51.2%, whereas it was 44.9% for Pap cytology [36]. Recent studies have proposed methylation biomarkers as potential tools for triage of hrHPV-positive women [37–42].

**Figure 3:**
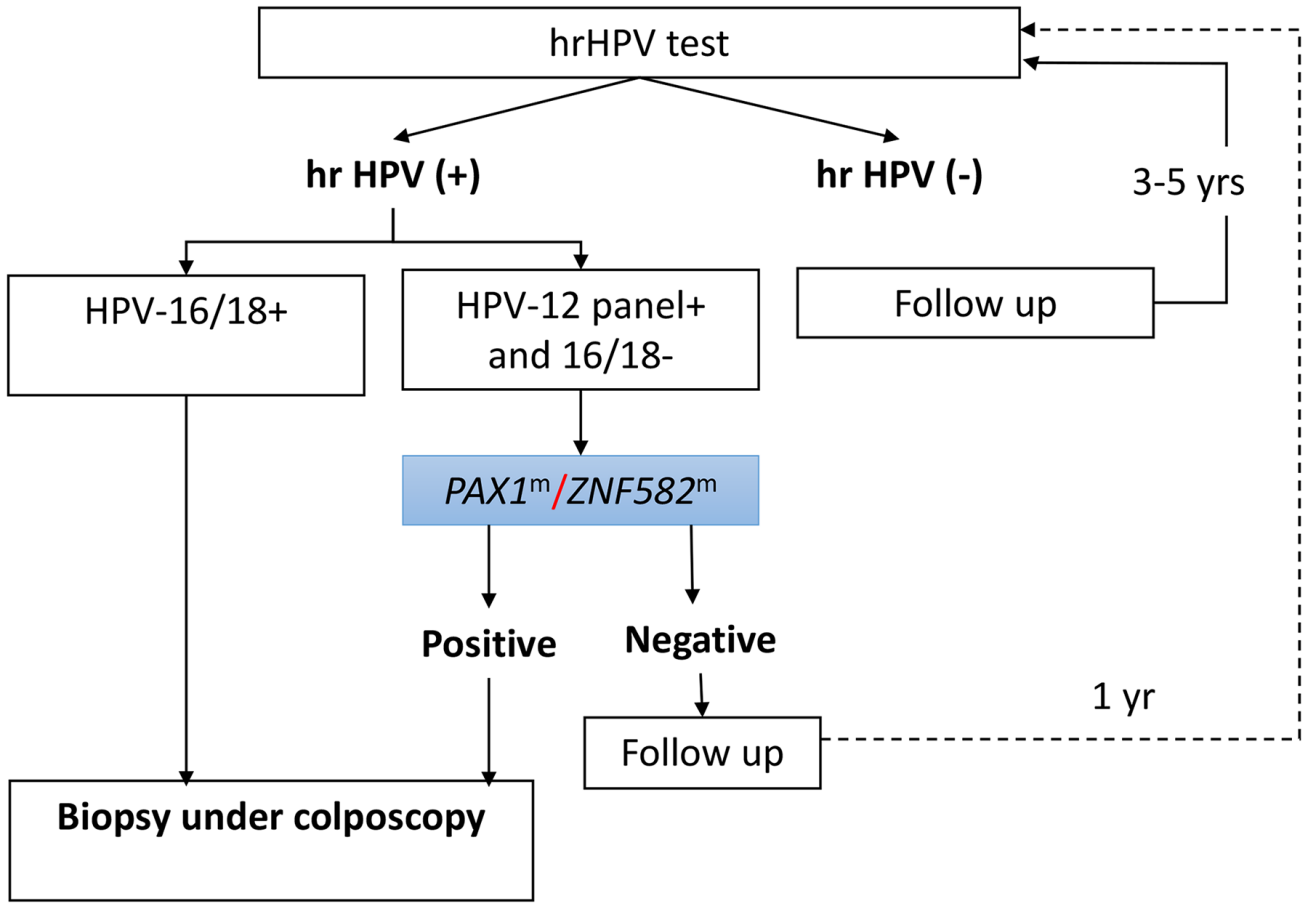
Proposed cervical cancer screening strategy using the hrHPV assay as a primary screening tool and testing for HPV-16/18 and methylation of *PAX1*/*ZNF582* as reflex triage tests In this proposed scenario, the hrHPV DNA assay is used as the primary screening test, and women without hrHPV infection undergo follow-up 3 to 5 years later. Samples from women with hrHPV infection undergo analysis of *PAX1* or *ZNF582* methylation and women positive for HPV-16/18 are referred for colposcopy. Patients positive for hrHPV types other than HPV-16/18 undergo testing for *PAX1* or *ZNF582* methylation. Patients with positive results for one of the two methylated genes are referred for colposcopy. Additionally, women testing positive for hrHPV but negative for methylation may undergo repeat HPV genotyping and DNA methylation analysis after 1 year.

There is increasing evidence that testing for methylated genes could replace cytology as a reflex test for women positive for the 12 hrHPV types other than HPV-16/18, and interim clinical guidance approves the use of such tests as an appropriate triage tool for hrHPV [13, 43]. We propose a cervical cancer screening strategy using the hrHPV genotyping assay and methylation analysis as triage tests (Figure 3). In hrHPV-positive patients, positive results in HPV-16/18 testing or gene methylation (*PAX1*^*m*^/*ZNF582*^*m*^) testing indicate a need for biopsy during colposcopy for diagnosis of CIN3+ lesions. We suggest a 1-year follow-up of patients with positivity for the 12 high-risk HPV genotypes but negative results for HPV-16/18 and methylated gene testing. Because HPV-16/18 and methylation co-testing demonstrated a 100% rate of positivity for cervical cancer identification in this study, we suggest that because of the lower risk for CIN3+ lesions, patients with negative results by hrHPV and methylated gene testing should be followed up after 5 years or more [39].

Several previous studies have supported the great promise of analysis of *PAX1*^*m*^ and *ZNF582*^*m*^ for detection of high-grade CIN lesions and cervical cancer. We explored the value of triage testing of hrHPV-positive women by combined testing for positivity for HPV-16/18 or the dual methylated markers, *PAX1*^*m*^ or *ZNF582*^*m*^. Combined triage resulted in substantially higher sensitivity and specificity for CIN3+, compared with cytology alone, genotyping for the 12 other hrHPV types individually, or cytology with HPV-16/18 testing. The strengths of the present study lie in its use of a delinked case-control study design and the incorporation of all histological results, including those of the normal cytology group.

In 2012, the ACS proposed new screening guidelines that included using age-adjusted screening methodology as well as cytological and HPV co-testing. HPV-16/18 typing has been recommended for hrHPV-positive women [44]; however, geographic variation exists in the distribution of HPV genotypes. In Asia, in addition to HPV types 16 and 18, types 52 and 58 are frequently observed in patients with invasive cervical cancers [2, 45-47]. Adoption of hrHPV testing will result in many more women becoming aware of their HPV status; however, this could result in psychological trauma or unnecessary anxiety about cervical cancer. The top five hrHPV genotypes identified in infections in 37 cities in China were HPV-16 (35.0%), HPV-52 (32.3%), HPV-58 (21.2%), HPV-59 (17.5%), and HPV-39 (17.2%) [47]. The proportion of these HPV genotypes that was associated with cervical cancer was HPV-16/18 (69.7%), HPV-58 (7.2%), HPV-52 (3.6%), HPV-33 (3.6%), and HPV-31 (2.3%); for other hrHPV types, the rates of cervical cancer were < 1% in a meta-analysis of data from Chinese women [3]. In the present study, the highest rates of cervical cancer were identified in women infected with hrHPV types HPV-16/18 (81%), HPV-58 (4.3%), HPV-52 (4.3%), HPV-31 (4.3%), and HPV-33 (1.4%), with other hrHPV types making up a further 4.3%. HPV types 52/58/31/33 made up 16.7% and 14.3% of cervical cancer diagnoses in the meta-analysis data and in our study, respectively. In the meta-analysis, HPV types 52/58/31/33 were detected in 48.0% of patients with LSIL and normal pathology, leading to an increase in false-positive rates when hrHPV testing was used as a triage tool.

The huge number of hrHPV patients referred for colposcopic examination and follow-up could be an extra burden in China, should HPV testing be used for hospital-based opportunistic screening. Our data demonstrate that following the current interim clinical guidance would not significantly reduce the referral rate for colposcopy. However, the substantial variability in sensitivity observed among areas with different cytology infrastructures and insufficient numbers of experienced cytologists are other factors reducing the referral rate for colposcopy in China. Comparison of cytology and dual methylated markers as reflex tests indicated that the dual methylated markers resulted in a referral rate for colposcopic examination that was 24.4% of the rate for cytology.

The present study has some potential limitations. Not all hrHPV-positive patients were referred for colposcopy examination because of the limited budget of the clinical study and based on the independent judgment of gynecologists in clinic. In addition, 144 hrHPV-ASC-US patient samples were collected during colposcopy because the majority of patients with obvious cervical cancer underwent biopsy immediately following abnormal Pap smear test results and clinical observation in the outpatient department. Other limitations include the small sample size and a lack of extensive and long-term follow-up information. In addition, the use of dual methylated markers as the only triage biomarker for hrHPV-positive women was not analyzed in this study, and this study is not representative of a typical screening population.

We recommend a new hrHPV flow-chart procedure for primary screening for cervical cancer. Our study suggests that the dual methylated markers, *PAX1*^*m*^ and *ZNF582*^*m*^, combined with HPV-16/18 testing, could help to reduce the number of hrHPV- positive women being referred for colposcopy. Furthermore, implementation of our recommendations could simplify the logistics of follow-up care, especially in regions where colposcopy is not readily available. We propose that the suggested approach would be useful in outpatient departments of hospitals in highly populated countries where screening is not readily accessible through community-based settings.

## MATERIALS AND METHODS

### Patient recruitment

In accordance with the Declaration of Helsinki, all patients provided informed consent for their participation in the clinical study protocol approved by the Institutional Review Board of the Department of Clinical Pharmacology at Xiangya Hospital, Central South University, China. The clinical trial was registered in the Chinese Clinical Trial Registry (ChiCTR-DOD-14005446). Study inclusion criteria were females who were sexually active and not pregnant, had an intact uterus, and had no history of treatment for cervical intraepithelial neoplasia (CIN) or cervical cancer. Patients who had a history of cancer related to the reproductive tract, therapy for cervical lesions, HPV vaccinations, or a current pregnancy were excluded. The recruited subjects were in attendance following referral for colposcopy examination, and consisted of patients with both normal Pap and abnormal Pap smear results, inflammation symptoms, cervical erosion, bleeding symptoms, or suspected cervical cancer.

The sub-group of patients who were hrHPV-positive was invited to the colposcopic examination room of the Department of Obstetrics & Gynecology to participate in a blinded study where testers were not provided with clinical data. After patients signed the informed consent form, each underwent a colposcopic examination and biopsy. The cytology results were classified according to the 2001 Bethesda System (TBS 2001). Colposcopy-directed biopsies were performed for histological analysis, according to standard procedures in China. The final diagnosis was based on the results of tissue-proven pathology. Standard guidelines for the management and treatment of cervical neoplasia were followed in all patients [47]. All patient recruitment and clinical information collection processes were periodically monitored, and Good Clinical Practice (GCP) guidelines were followed.

In total, 461 patients were enrolled in the study from November 2011 to March 2013. Twelve patients were excluded based on the exclusion criteria, including five who were excluded because of poor-quality DNA specimens. This study focusses on data from the sub-group of 312 women who were hrHPV-positive following interim clinical guidance [13].

### Specimen collection and DNA preparation

All liquid-based cytology samples were clinician-collected using the Cytoprep Brush (Hospitex Diagnostics SRL, Sesto Fiorentino, Italy) and preserved in CytoFast Solution (Hospitex Diagnostics SRL, Sesto Fiorentino, Italy) in the clinic one week prior to the colposcopy visit. Residual cervical cells from cytological tests were used for HPV genotyping and methylation detection tests for the four genes. All specimens collected were given an identification number and delinked from patient clinical information until final data analysis. All molecular tests were performed at the Institute of Clinical Pharmacology, Hunan Key Laboratory of Pharmacogenetics, China, following Good Laboratory Practice (GLP) guidelines. The cells were centrifuged and stored in phosphate-buffered saline at -20°C from the day of collection. Genomic DNA (gDNA) was extracted from the collected cells using the QIAamp DNA Mini Kit (Qiagen GmbH, Hilden, Germany). A BioSpec-nano spectrophotometer (Shimadzu Corporation, Tokyo, Japan) was used to quantify the amount of extracted DNA.

### DNA methylation tests

Briefly, 500 ng of gDNA was subject to bisulfite conversion using EZ DNA Methylation-Gold kits (Zymo Research, Irvine, CA, USA). The methylation levels of *PAX1*, *ZNF582*, *SOX1*, and *NKX6-1* were determined using the iStat Biomedical Q-PCR kit on the LightCycler LC480 real-time PCR system (Roche Applied Science, Penzberg, Germany), according to the manufacturer’s protocol. The criteria for positive and negative results for the methylated genes were based on the delta crossing point (Cp) values, as determined in previous studies [20, 23, 25].

### Laboratory methods for HPV DNA amplification and genotyping

The hrHPV typing testing (EpiGene HPV-M SpeedGel Kit; iStat Biomedical Co., Ltd., Taiwan) was a clinical test performed using a nested multiplex PCR assay that combined degenerate E6/E7 consensus primers and type-specific primers as previously described [48, 49]. The hrHPV genotype was determined according to the size of the nested PCR amplification product.

### Statistical analyses

The positive cutoff values for methylated genes were determined as *PAX1*^*m*^ = ΔCp ≤ 9, *SOX1*^*m*^ = ΔCp ≤ 8, *ZNF582*^*m*^ = ΔCp ≤ 11, and *NKX6-1*^*m*^ = ΔCp ≤ 11. SPSS software (version 16.0, Chicago, IL, USA) was used for all statistical analyses. Both Chi-squared and Fisher’s exact tests were used to analyze the status of the methylated genes or of HPV genotyping in different combinations. Fisher’s exact test is considered more accurate than the chi-squared test when the sample size is smaller than five. A cross-validated receiver operating characteristic (ROC) curve was generated and the area under the ROC curve (AUC) for CIN3+ lesions was calculated for each detection method. The sensitivity, specificity, and odds ratio (OR) with 95% confidence interval (CI) were calculated for lesions classified as CIN3 or worse. All tests were two-sided and differences were considered statistically significant at *P* < 0.05.

## References

[1] Tota JE, Ramana-Kumar AV, El-Khatib Z, Franco EL (2014). The road ahead for cervical cancer prevention and control. Curr Oncol.

[2] Huang YK, You SL, Yuan CC, Ke YM, Cao JM, Liao CY, Wu CH, Hsu CS, Huang KF, Lu CH, Twu NF, Chu TY (2008). Long-term outcomes of high-risk human papillomavirus infection support a long interval of cervical cancer screening. Br J Cancer.

[3] Bao YP, Li N, Smith JS, Qiao YL (2008). Human papillomavirus type-distribution in the cervix of Chinese women: a meta-analysis. Int J STD AIDS.

[4] Tjalma WA, van Waes TR, van den Eeden LE, Bogers JJ (2005). Role of human papillomavirus in the carcinogenesis of squamous cell carcinoma and adenocarcinoma of the cervix. Best Pract Res Clin Obstet Gynaecol.

[5] Jemal A, Simard EP, Dorell C, Noone AM, Markowitz LE, Kohler B, Eheman C, Saraiya M, Bandi P, Saslow D, Cronin KA, Watson M, Schiffman M (2013). Annual Report to the Nation on the Status of Cancer, 1975–2009, featuring the burden and trends in human papillomavirus (HPV)-associated cancers and HPV vaccination coverage levels. J Natl Cancer Inst.

[6] Kim JJ, Campos NG, Sy S, Burger EA, Cuzick J, Castle PE, Hunt WC, Waxman A, Wheeler CM (2015). Inefficiencies and high-value improvements in U.S. cervical cancer screening practice: a cost-effectiveness analysis. Ann Intern Med.

[7] Brotherton JM, Gertig DM, May C, Chappell G, Saville M (2016). HPV vaccine impact in Australian women: ready for an HPV-based screening program. Med J Aust.

[8] Smith M, Canfell K (2016). Impact of the Australian National Cervical Screening Program in women of different ages. Med J Aust.

[9] Saslow D, Solomon D, Lawson HW, Killackey M, Kulasingam SL, Cain J, Garcia FA, Moriarty AT, Waxman AG, Wilbur DC, Wentzensen N, Downs LS, Spitzer M (2012). ACS-ASCCP-ASCP Cervical Cancer Guideline Committee. American Cancer Society, American Society for Colposcopy and Cervical Pathology, and American Society for Clinical Pathology screening guidelines for the prevention and early detection of cervical cancer. CA Cancer J Clin.

[10] Gage J, Katki HA, Schiffman M, Castle PE, Fetterman B, Wentzensen N, Poitras NE, Lorey T, Cheung LC, Kinney WK (2014). Reassurance against future risk of precancer and cancer conferred by a negative human papillomavirus test. J Natl Cancer Inst.

[11] Wright TC, Stoler MH, Behrens CM, Sharma A, Zhang G, Wright TL (2015). Primary cervical cancer screening with human papillomavirus: end of study results from the ATHENA study using HPV as the first-line screening test. Gynecol Oncol.

[12] Ronco G, Giorgi-Rossi P, Carozzi F, Carozzi F, Confortini M, Dalla Palma P, Del Mistro A, Gillio-Tos A, Minucci D, Naldoni C, Rizzolo R, Schincaglia P, Volante R (2008). Results at recruitment from a randomized controlled trial comparing human papillomavirus testing alone with conventional cytology as the primary cervical cancer screening test. J Natl Cancer Inst.

[13] Huh WK, Ault KA, Chelmow D, Davey DD, Goulart RA, Garcia FA, Kinney WK, Massad LS, Mayeaux EJ, Saslow D, Schiffman M, Wentzensen N, Lawson HW (2015). Use of primary high-risk human papillomavirus testing for cervical cancer screening: interim clinical guidance. Gynecol Oncol.

[14] Practice Bulletin No 157 (2016). Cervical Cancer Screening and Prevention. Obstet Gynecol.

[15] Stewart BW, Wild CP

[16] Wang R, Guo XL, Wisman GB, Schuuring E, Wang WF, Zeng ZY, Zhu H, Wu SW (2015). Nationwide prevalence of human papillomavirus infection and viral genotype distribution in 37 cities in China. BMC Infect Dis.

[17] Farkas SA, Milutin-Gasperov N, Nilsson TK (2014). Genome-wide DNA methylation assay reveals novel candidate biomarker genes in cervical cancer. Epigenetics.

[18] Lendvai Á, Johannes F, Grimm C, Eijsink JJ, Wardenaar R, Volders HH, Klip HG, Hollema H, Jansen RC, Schuuring E, Wisman GB, van der Zee AG (2012). Genome-wide methylation profiling identifies hypermethylated biomarkers in high-grade intraepithelial neoplasia. Epigenetics.

[19] Wentzensen N, Sherman ME, Schiffman M, Wang SS (2009). Utility of methylation markers in cervical cancer early detection: appraisal of the state-of-the-science. Gynecol Oncol.

[20] Kan YY, Liou YY, Wang HJ, Chen CY, Sung LC, Chang CF, Liao CI (2014). PAX1 methylation as a potential biomarker for cervical cancer screening. Int J Cancer.

[21] Lai HH, Qu YC, Chen TC, Huang HJ, Cheng YM, Chen CH, Chu TY, Hsu ST, Liu CB, Hung YC, Wen KC, Yu MH, Wang KL (2014). PAX1/SOX1 DNA methylation and cervical neoplasia detection: a Taiwanese Gynecologic Oncology group (TGOG) study. Cancer Med.

[22] Lin H, Chen TC, Chang TC, Cheng YM, Chen CH, Chu TY, Hsu ST, Liu CB, Yeh LS, Wen KC, Huang CY, Yu MH (2014). Methylated ZNF582 gene as a marker for triage of women with Pap smear reporting low grade squamous intraepithelial lesions- a Taiwanese Gynecologic Oncology Group (TGOG) study. Gynecol Oncol.

[23] Liou YL, Zhang Y, Liu Y, Cao L, Qin CZ, Zhang TL, Chang CF, Wang HJ, Lin SY, Chu TY, Zhang Y, Zhou HH (2015). Comparison of HPV genotyping and methylated ZNF582 as triage for women with equivocal liquid-based cytology results. Clin Epigenetics.

[24] Pun PB, Liao YP, Su PH, Wang HC, Chen YC, Hsu YW, Huang RL, Chang CC, Lai HC (2015). Triage of high-risk human papillomavirus-positive women by methylated POU4F3. Clin Epigenetics.

[25] Liou YL, Zhang TL, Yan T, Yeh CT, Kang YN, Cao L, Wu N, Chang CF, Wang HJ, Yen C, Chu TY, Zhang Y, Zhang Y, Zhou H (2016). Combined clinical and genetic testing algorithm for cervical cancer diagnosis. Clin Epigenetics.

[26] Lai HC, Lin YW, Huang RL, Chung MT, Wang HC, Liao YP, Su PH, Liu YL, Yu MH (2010). Quantitative DNA methylation analysis detects cervical intraepithelial neoplasms type 3 and worse. Cancer.

[27] Huang RL, Chang CC, Su PH, Chen YC, Liao YP, Wang HC, Yo YT, Chao TK, Huang HC, Lin CY, Chu TY, Lai HC (2012). Methylomic analysis identifies frequent DNA methylation of zinc finger protein 582 (ZNF582) in cervical neoplasms. PLoS One.

[28] Chang CC, Huang RL, Wang HC, Liao YP, Yu MH, Lai HC (2014). High methylation rate of LMX1A, NKX6-1, PAX1, PTPRR, SOX1, and ZNF582 genes in cervical adenocarcinoma. Int J Gynecol Cancer.

[29] Bosgraaf RP, Verhoef VM, Massuger LF, Siebers AG, Bulten J, de Kuyper-de Ridder GM, Meijer CJ, Snijders PJ, Heideman DA, IntHout J, van Kemenade FJ, Melchers WJ, Bekkers RL (2015). Comparative performance of novel self-sampling methods in detecting high-risk human papillomavirus in 30,130 women not attending cervical screening. Int J Cancer.

[30] Chang CC, Huang RL, Liao YP, Su PH, Hsu YW, Wang HC, Tien CY, Yu MH, Lin YW, Lai HC (2015). Concordance analysis of methylation biomarkers detection in self-collected and physician-collected samples in cervical neoplasm. BMC Cancer.

[31] Lorincz A, Castanon A, Lim AW (2013). New strategies for HPV-based cervical screening. Womens Health (Lond Engl).

[32] Hansel A, Steinbach D, Greinke C, Schmitz M, Eiselt J, Scheungraber C, Gajda M, Hoyer H, Runnebaum IB, Dürst M (2014). A promising DNA methylation signature for the triage of high-risk human papillomavirus DNA-positive women. PLoS One.

[33] Cuzick J (2010). long-term follow-up in cancer prevention trials (It ain’t over ‘til it’s over). Cancer Prev Res (Phila).

[34] De Strooper LM, Hesselink AT, Berkhof J, Meijer CJ, Snijders PJ, Steenbergen RD, Heideman DA (2014). Combined CADM1/MAL methylation and cytology testing for colposcopy triage of high-risk HPV-positive women. Cancer Epidemiology Biomark Prev.

[35] Wentzensen N, Schwartz L, Zunaet RE, Smith K, Mathews C, Gold MA, Allen RA, Zhang R, Dunn ST, Walker JL, Schiffman M (2012). Performance of p16/Ki-67 immunostaining to detect cervical cancer precursors in a colposcopy referral population. Clin Cancer Res.

[36] Luttmer R, Dijkstra MG, Snijders P, Berkhof J, van Kemenade FJ, Rozendaal L, Helmerhorst TJ, Verheijen RH, Ter Harmsel WA, van Baal WM, Graziosi PG, Quint WG, Spruijt JW (2016). p16/Ki-67 dual-stained cytology for detecting cervical (pre)cancer in a HPV-positive gynecologic outpatient population. Mod Pathol.

[37] Overmeer RM, Louwers JA, Meijer CJ, van Kemenade FJ, Hesselink AT, Daalmeijer NF, Wilting SM, Heideman DA, Verheijen RH, Zaal A, van Baal WM, Berkhof J, Snijders PJ (2011). Combined CADM1 and MAL promoter methylation analysis to detect (pre-)malignant cervical lesions in high-risk HPV-positive women. Int J Cancer.

[38] Hesselink AT, Heideman DA, Steenbergen RD, Coupé VM, Overmeer RM, Rijkaart D, Berkhof J, Meijer CJ, Snijders PJ (2011). Combined promoter methylation analysis of CADM1 and MAL: an objective triage tool for high-risk human papillomavirus DNA-positive women. Clin Cancer Res.

[39] Eijsink JJ, Lendvai A, Deregowski V, Klip HG, Verpooten G, Dehaspe L, de Bock GH, Hollema H, van Criekinge W, Schuuring E, van der Zee AG, Wisman GB (2012). A four-gene methylation marker panel as triage test in high-risk human papillomavirus positive patients. Int J Cancer.

[40] Vasiljevic N, Scibior-Bentkowska D, Brentnall AR, Cuzick J, Lorincz AT (2014). Credentialing of DNA methylation assays for human genes as diagnostic biomarkers of cervical intraepithelial neoplasia in high-risk HPV positive women. Gynecol Oncol.

[41] Pun PB, Liao YP, Su PH, Wang HC, Chen YC, Hsu YW, Huang RL, Chang CC, Lai HC (2015). Triage of high-risk human papillomavirus-positive women by methylated POU4F3. Clin Epigenetics.

[42] Boers A, Wang R, van Leeuwen RW, Klip HG, de Bock GH, Hollema H, van Criekinge W, de Meyer T, Denil S, van der Zee AG, Schuuring E, Wisman GB (2016). Discovery of new methylation markers to improve screening for cervical intraepithelial neoplasia grade 2/3. Clin Epigenetics.

[43] Luttmer R, De Strooper LM, Berkhof J, Snijders PJ, Dijkstra MG, Uijterwaal MH, Steenbergen RD, van Kemenade FJ, Rozendaal L, Helmerhorst TJ, Verheijen RH, Ter Harmsel WA, Van Baal WM (2016). Comparing the performance of FAM19A4 methylation analysis, cytology and HPV16/18 genotyping for the detection of cervical (pre)cancer in high-risk HPV-positive women of a gynecologic outpatient population (COMETH study). Int J Cancer.

[44] Wright T (2016). Huang J1, Baker E, Garfield S, Hertz D, Cox JT. The budget impact of cervical cancer screening using HPV primary screening. Am J Manag Care.

[45] Li H, Zhang J, Chen Z, Zhou B, Tan Y (2013). Prevalence of human papillomavirus genotypes among women in Hunan province, China. Eur J Obstet Gynecol Reprod Biol.

[46] Quek SC, Lim BK, Domingo E, Soon R, Park JS, Vu TN, Tay EH, Le QT, Kim YT, Vu BQ, Cao NT, Limson G, Pham VT (2013). Human papillomavirus type distribution in invasive cervical cancer and high-grade cervical intraepithelial neoplasia across 5 countries in Asia. Int J Gynecol Cancer.

[47] Wang R, Guo XL, Wisman GB, Schuuring E, Wang WF, Zeng ZY, Zhu H, Wu SW (2015). Nationwide prevalence of human papillomavirus infection and viral genotype distribution in 37 cities in China. BMC Infect Dis.

[48] Saslow D, Solomon D, Lawson HW, Killackey M, Kulasingam SL, Cain J, Garcia FA, Moriarty AT, Waxman AG, Wilbur DC, Wentzensen N, Downs LS, Spitzer M (2012). American Cancer Society, American Society for Colposcopy and Cervical Pathology, and American Society for Clinical Pathology screening guidelines for the prevention and early detection of cervical cancer. Am J Clin Pathol.

[49] Lin H, Moh JS, Ou YC, Shen SY, Tsai YM, ChangChien CC, Liu JM, Ma YY (2005). A simple method for the detection and genotyping of high-risk human papillomavirus using seminested polymerase chain reaction and reverse hybridization. Gynecol Oncol.

